# A case report on a rare cause of bowel ischaemia in penetrating trauma

**DOI:** 10.1016/j.ijscr.2021.106010

**Published:** 2021-05-25

**Authors:** Chido Nyatsambo, Krevosha Pillay, Maeyane Steve Moeng, Michael John Savage-Reid, Megan Lubout

**Affiliations:** a Division of Trauma, Department of General Surgery, Charlotte Maxeke Johannesburg Academic Hospital, 5 Jubilee Street, Parktown, Johannesburg, South Africa; b Division of Anatomical Pathology, National Health Laboratory Service, Charlotte Maxeke Johannesburg Academic Hospital, 5 Jubilee Street, Parktown, Johannesburg, South Africa

**Keywords:** Penetrating trauma, Gastric mucormycosis, Bowel ischaemia

## Abstract

**Introduction and importance:**

Gastric mucormycosis is a rare condition that usually manifests in immunocompromised patients. It's a lethal disease with a poor prognosis requiring prompt diagnosis and aggressive management. Although found more commonly in immunocompromised patients, it can also affect the immunocompetent patient, highlighting the importance of clinical suspicion when dealing with a critically ill patient.

**Case presentation:**

This is a case report on a patient who presented with penetrating trauma to the abdomen requiring surgical intervention. Damage control surgery was performed in the form of a right hemicolectomy (‘clip and drop’) for extensive colonic injuries (AAST Grade V) with contamination of the abdominal cavity [1]. In the days subsequent to the injury, he developed sepsis and progressive bowel ischaemia and necrosis, requiring surgical debridement. Histological findings revealed mucormycosis of the gastrointestinal tract.

**Clinical discussion:**

The diagnosis of mucormycosis depends on high clinical suspicion as well as histopathological evidence. The management comprises of surgical debridement and appropriate antifungal therapy. Timeous diagnosis and adequate treatment may improve the prognosis.

**Conclusion:**

This was a challenging case for the clinicians involved, highlighting that the clinician should consider this infection as a rare cause of bowel ischaemia in the back of their minds when dealing with such patients.

## Highlights

• Emphasizing the need for a high clinical suspicion & diagnosis of rare intra-abdominal infections (gastric mucormycosis)
• Identification of an unusual cause of progressive bowel ischaemia in a critically ill patient admitted to the ICU
• Management principles of gastric mucormycosis centred around prompt diagnosis, antifungal therapy and surgical debridement.
• A reminder of unusual abdominal complication seen in penetrating trauma

## Graphical abstract

**Figure:**
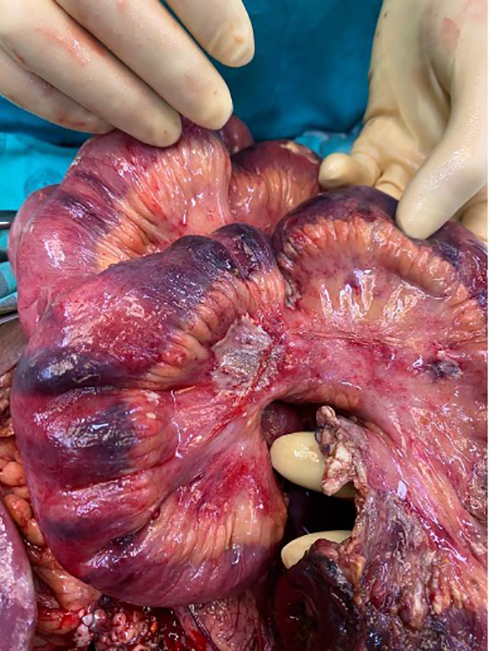

## 1 Introduction

Mucormycosis is a rare fungal infection arising from the zygomycetes species [2]. It affects patients with the following risk factors: acidosis, uncontrolled diabetes mellitus, diabetic ketoacidosis, septicaemia, leukaemia, lymphoma, HIV/AIDS, severe malnourishment, severe burns, cytotoxic therapy, immunosuppression from corticosteroids [2,3]. Other risk factors include chronic renal failure, liver pathology and dialysis patients receiving deferoxamine [2,4]. Treatment of mucormycosis is centred around surgical debridement of the necrotic tissue and administration of a lipid-based formulation of amphotericin B [3]. This case report identifies a rare case of bowel ischemia after penetrating intra-abdominal trauma. Only a few cases of this condition in trauma are reported, most of whom presented with GIT bleeding [[4], [5], [6], [7], [8], [9], [10]]. This case report has been written in line with the SCARE guidelines [11].

## 2 Case presentation

A 30-year-old man presented to our Trauma unit with penetrating abdominal trauma. He had no known background medical or surgical history. Of note were two gunshot wounds - one thoracoabdominal in the 8th intercostal space on the right and a second wound over the right buttock. He came in haemodynamically unstable with a blood pressure of 60/40 mmHg pulse of 100 beats per minute. He was resuscitated according to the ATLS® principles of Primary and Secondary survey. The arterial blood gas showed severe metabolic acidosis. Fluid resuscitation and massive transfusion protocol were initiated per standard of care. The patient was taken to theatre for an emergency damage control laparotomy by the Trauma surgeon and a senior trauma registrar (resident).

Haemoperitoneum, gross faecal contamination and multiple hollow viscus injuries were confirmed at laparotomy. Bleeding from the bowel mesentery and the bowel ends was controlled first, without any overt bowel blood supply changes. Minor contusion of the second part of the duodenum (AAST Grade I) was noted at laparotomy, requiring no intervention [1]. Extensive caecal and transverse colon injuries (AAST Grade V), not amenable to repair, were noted. A right hemicolectomy was performed with a GIA stapler resulting in the typical ‘clip-and drop’ of the bowel ends [1]. The patient was transfused four units of packed red blood cells, four fresh frozen plasma units, and one mega unit of platelets intra-operatively. The abdomen was left open with a temporary vacuum-assisted abdominal closure, with a plan to return to theatre for definitive surgery.

He was transferred postoperatively to the trauma intensive care unit (ICU) for continued resuscitation and close monitoring. Given gross contamination, broad-spectrum antibiotics were continued in ICU. He still required vasoactive support to maintain acceptable mean arterial pressures. His acidosis corrected within 24 h of his ICU admission, although he still had features of Systematic Inflammatory Response Syndrome (SIRS) as evidenced by ongoing pyrexia and persistent tachycardia. A planned relook laparotomy was performed on day 2. The abdomen was noted to be clean at relook. The transverse colon stump was intact, all the remaining bowel was viable, and an end ileostomy was fashioned.

Day 3 post-operation, he developed an acute kidney injury and mild hypoglycaemia attributed to possible systemic sepsis. His antibiotics were escalated to a carbapenem as per the trauma unit protocol. A septic workup (blood cultures, Chest X-rays etc.) was done, including a 1,3 Beta D glucan assay (Fungitell®). The BDG result was significantly raised (500 pg/ml), highly suggestive of invasive fungal sepsis. As per our local micro-biogram, he was started on an antifungal cover using Fluconazole at 800 mg daily. He showed good clinical improvement and was successfully weaned off a ventilator and extubated on day 5 post-operation.

**Fig. 1:**
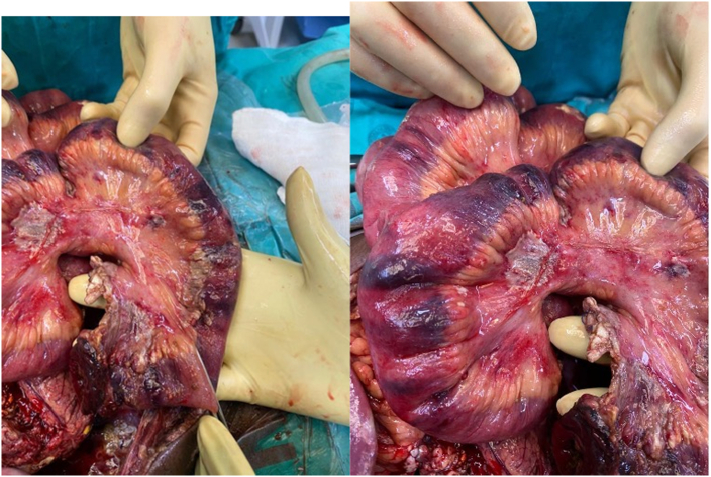
a and b: intra-operative findings of small bowel showing areas of patchy necrosis.

Previously well-perfused ileostomy became necrotic on Day 7 post-operation. At this stage, the patient had been off vasoactive medication for more than 5 days. A relook on demand was performed in theatre. Multiple segments of patchy necrosis and ischaemia starting 160 cm from the duodenal jejunal flexure (Fig. 1a, b) were discovered. No surgical, mechanical explanation or complications could be elicited for the cause of bowel ischaemia. This finding was unusual and surprised the surgical team. Small bowel resection of necrotic bowel was done, and specimens sent off for histology. Unfortunately, in our setting, the frozen section is not available after hours. The necrosis was associated with features of early perforation in some regions.

**Fig. 2:**
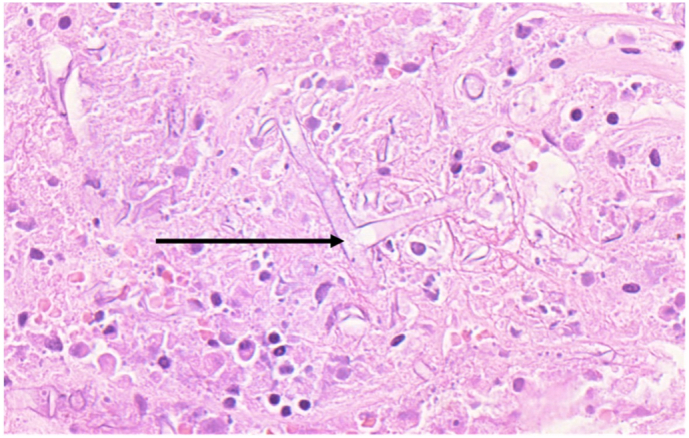
H&E, 400×; broad, non-septate hyphae with 90 angle branching (arrow) and background necrotic debris.

**Fig. 3:**
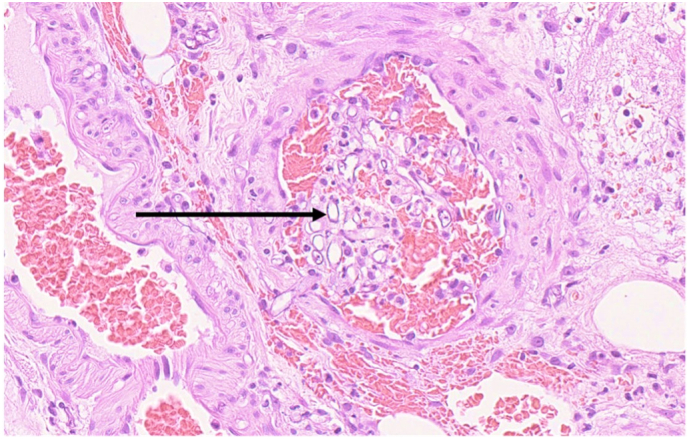
H&E, 200×; fungal hyphae within both a muscular vessel wall and lumen (arrow), demonstrating angioinvasion.

The pathology report confirmed a diagnosis of mucormycosis on the segment of resected small bowel that was submitted for histopathological examination. Serosal fibrinopurulent exudate and a site of perforation were identified macroscopically and features of haemorrhagic infarction. Haemotoxylin and eosin (H&E) sections were representative of small bowel wherein transmural necrosis, perforation and acute serositis were confirmed. Large areas of mucosal necrosis containing fungal hyphae were present (Fig. 2). The hyphae were large, non-septate, lacked parallel walls and demonstrated acute angle branching, including 90-degree angle branching, morphologically consistent with mucormycosis. Transmural invasion by the hyphae with extensive angioinvasion was also demonstrated (Fig. 3).

At this stage, we had histological evidence of mucormycosis. This was despite negative screening for common associated medical conditions in this patient. Workup for Diabetes mellitus, Tuberculosis, Human immune deficiency virus and hypertension were all negative. There were no immunosuppressive medications identified in the history provided or administered during his stay with us before this development. The patient continued to have a turbulent course and required a further two relooks. He demised despite additional antimicrobial support and escalating antifungal therapy to include Amphotericin B therapy. Low dose physiological intravenous steroids were introduced for refractory hypotension towards the end.

## 3 Discussion

Mucormycosis is an angioinvasive fungal infection arising from the zygomycetes species [2]. The organism is found in the environment on decaying vegetation and soil. Fungi are common organisms, and humans have day to day contact with them [12]. The intact innate immune system of an individual prevents the formation of various infections in the human body; however, in immunocompromised patients, especially diabetic patients, this organism can present in various syndromes. Zygomycetes has two orders: Entomophthorales and Mucorales. The latter being implicated in causing the infection, specifically, *Rhizopus*, *Mucor*, and *Rhizomucor* [13].

There seems to be an increase in the number of mucormycosis cases reported. A review of case reports in South America shows that the number of reported cases has increased since 1970 [14]. The overall mortality has been quoted to be up to 48% in some case series reviews and up to 70% in others [2,14]. Risk factors are well described, as already mentioned in the introduction [[2], [3], [4]]. In 19% of cases, no underlying condition can be found [6]. Our patient had no other risk factors apart from severe trauma resulting in acidosis. The use of steroids for refractory hypotension was only used later and at physiological doses.

Though the usual site involves paranasal sinuses in 39% of the cases, GIT involvement has been reported in 7% [16]. The fungi have a preference for arterial invasion, where they cause extensive emboli and necrosis of surrounding tissues resulting in ischemia and necrosis of the affected tissue [2,4]. This manifests as ulceration, necrosis and infarction of the affected tissue [13]. If the GIT is affected, patients can present with upper gastrointestinal bleed, bowel perforation and even bowel ischaemia.

To make the diagnosis, the presence of predisposing conditions, signs and symptoms, and tissue specimens are required. Surgical specimens taken intraoperatively or via endoscopy should be analysed with direct microscopy and histopathology. Culture, while considered essential for identification and antimicrobial susceptibility, has a low sensitivity [16]. There are no serological or PCR tests that are diagnostic for mucormycosis at present [4,17]. Autopsy series have demonstrated that mucormycosis is often diagnosed postmortem – especially in the gastrointestinal tract infection [2,17].

We feel that the development of bowel necrosis when the patient was already off vasoactive drugs is suggestive of the contribution of mucormycosis to ischaemia in this case. No ischaemia had been noted on the prior laparotomy procedures. Though our patient did not present with gastrointestinal bleeding, the case highlights the difficulties of diagnosing this condition in trauma.

There are four management principles: prompt diagnosis, optimisation of risk factors, early initiation of appropriate antifungal therapy, and surgical debridement [17]. Immuno-suppressive medications (steroids in particular) should be stopped wherever possible, and hyperglycaemia and acidosis should be corrected. There are no prospective randomised trials to define the optimal antifungal treatment for mucormycosis [17]. Improved survival is noted with the initiation of antifungals within five days [4].

We, unfortunately, used the azoles to manage him initially before the histology report was made available to us. In our setting, most fungal sepsis does respond to azoles as resistance has not yet reached levels seen in other centres unless otherwise stated by the centres biogram [18]. The azoles are ineffective when treating Mucormycosis, except Posaconazole or Isavuconazole, which may be used in refractory mucormycosis [6,17]. Global guidelines recommend liposomal amphotericin B 5-10 mg/kg per day as first-line treatment for any site involved; duration of therapy being individualized until clearance of infection [19]. Resistance to amphotericin B has been noted, especially with prolonged therapy [2,17]. Angioinvasion, thrombosis and tissue necrosis also result in poor penetration of antifungal agents, necessitating extensive surgical debridement of necrotic tissue [4,6,17]. However, in patients who have the non-invasive form of the infection with no features of necrosis or thrombosis, medical therapy alone has found to be effective in some cases.

## 4 Conclusion

This is an unusual case of fungal sepsis in penetrating trauma. It is a rare condition found in immunocompromised patients. However, even with the lack of significant risk factors other than acidosis, the clinician should be alerted to a possibility of this diagnosis when dealing with unexplained bowel ischaemia. We will certainly think about this diagnosis for any unexplained bowel ischaemic cases in the future, as appropriate antifungal therapy and debridement remain the cornerstone of care of this condition which is associated with high mortality.

## Funding

This research did not receive any specific grant from funding agencies in the public, commercial, or not-for-profit sectors.

## Ethical approval

The University of Witwatersrand Johannesburg Human Research Ethics Committee (medical) approved this case report (M2011138).

## Consent

Written informed consent was not obtained from the patient, as the patient demised. The head of the Department of Surgery and our medical team has taken responsibility for exhaustive attempts to contact the family. The paper has been sufficiently anonymized to avoid harm to the patient and the family. A signed document stating the above is available for review by the Editor-in-chief of this journal upon request.

## CRediT authorship contribution statement

All authors wrote the case report. Dr. C Nyatsambo and Professor Moeng organized the manuscript and revised the paper.

## Research registration

Not applicable.

## Guarantor

Professor MS Moeng.

## Provenance and peer review

Not commissioned, externally peer-reviewed.

## Declaration of competing interest

None.
